# Prognostic factors for acute kidney injury development in critically ill cancer patients

**DOI:** 10.1186/cc10181

**Published:** 2011-06-22

**Authors:** LA Hajjar, H Palomba, J Almeida, J Fukushima, RE Nakamura, F Galas, V Torres, R Kalil Filho, L Yu

**Affiliations:** 1 ICESP, São Paulo - SP, Brazil

## Introduction

Acute kidney injury (AKI) in cancer patients is a complication that causes substantial morbidity and mortality.

## Methods

A total of 1,500 cancer patients admitted to the medical intensive care unit (ICU) between November 2008 and February-March 2011 were evaluated for AKI, defined as an increase in serum creatinine (SCr) >0.3 mg/dl over the baseline value, according to the AKIN stage I definition. Univariate analysis was used to study ICU admission parameters associated with AKI occurrence during the ICU stay.

## Results

AKI incidence was 31%, with a mortality rate of 42%, compared with 20% for non-AKI patients. The mean age of all patients was 63.1 ± 11.3 years and 55% were male. Sepsis (44.8%) and respiratory failure (24.8%) were the major reasons for admission to the ICU. At univariate analysis, the following variables at ICU admission were significantly associated with AKI in cancer patients during the ICU stay: need for vasopressors (74.3% vs. 25.7%; *P *= 0.004), serum potassium (4.2, 3.6 to 4.6 mEq/l vs. 3.8, 3.5 to 4.2 mEq/l; *P *= 0.006), serum pH (7.35, 7.3 to 7.39 vs. 7.39, 7.34 to 7.42; *P *= 0.006), base excess (-5.5, -9.2 to -1.8 vs. -2, -5 to 0.1; *P *= 0.003), serum phosphorus (3.9, 3.4 to 4.6 mg/dl vs. 2.9, 2.4 to 3.9 mg/dl; *P *= 0.0001), baseline serum creatinine (1.2, 0.7 to 1.8 mg/dl vs. 0.6, 0.4 to 0.8 mg/dl; *P *= 0.01). At multivariate analysis, the following variables at ICU admission were associated with AKI: serum creatinine >1.0 mg/dl (OR = 9.2; 95% CI = 2.3 to 35.8), pH <7.38 (OR = 5.1; 95% CI = 1.6 to 15.6) and need for vasopressors in the first 24 hours (OR = 3.4; 95% CI = 1.2 to 9.6). Variables previously thought to be indicative of a poor prognosis (advanced age, metastatic or progressive disease, recent chemotherapy and performance status) were not predictive of AKI.

## Conclusions

AKI is frequent in critically ill cancer patients and has a great impact on mortality. AKI incidence can be better estimated by an evaluation of the acute organ dysfunction at ICU admission than by the characteristics of the underlying malignancy.

